# Serological Profiles of Hepatitis B Virus in Patients With Crohn’s Disease Undergoing Anti-Tumor Necrosis Factor Alpha (TNFα) Therapy at Ibn Sina University Hospital, Rabat

**DOI:** 10.7759/cureus.73550

**Published:** 2024-11-12

**Authors:** Imane Louhab, Hajar Daoudi, Mina Elcadi, Ghizlane El Amin, Amal Zouaki, Jalila Zirar, Myriam Seffar, Mouna Salihoun, Hakima Kabbaj

**Affiliations:** 1 Central Laboratory of Hematology, Ibn Sina University Hospital, Faculty of Medicine and Pharmacy, Mohammed V University, Rabat, MAR; 2 Department of Pharmacy, Ibn Sina University Hospital, Faculty of Medicine and Pharmacy, Mohammed V University, Rabat, MAR; 3 Central Laboratory of Virology, Ibn Sina University Hospital, Faculty of Medicine and Pharmacy, Mohammed V University, Rabat, MAR; 4 Department of Gastroenterology, Ibn Sina University Hospital, Faculty of Medicine and Pharmacy, Mohammed V University, Rabat, MAR

**Keywords:** anti-tnfα, crohn’s disease (cd), hbv serological markers, hepatitis b virus reactivation, viral hepatitis b

## Abstract

Introduction

Anti-tumor necrosis factor alpha (TNFα) therapies have revolutionized the management of Crohn’s disease (CD). However, they increase the risk of viral reactivation, particularly hepatitis B virus (HBV). This study aims to define the HBV serological profiles of patients with CD who are candidates for biological therapy, identifying profiles at potential risk for reactivation or exacerbation following immunosuppressive treatment.

Materials and methods

This descriptive retrospective study included patients with CD, aged over 16 years, who were candidates for anti-TNFα treatment at Ibn Sina University Hospital Center (UHC) in Rabat, Morocco, from January 2015 to March 2023. The serological profiles of patients, including hepatitis B surface antigen (HBsAg), hepatitis B surface antibodies, and total hepatitis B core antibodies (HBcAb), were determined using microparticle chemiluminescence immunoassay with the ARCHITECT i2000sr or Alinity (Abbott Diagnostics, Chicago, Illinois, United States) automated systems at the Central Virology Laboratory (CVL) of Ibn Sina UHC Rabat. HBV DNA quantification was performed using the m2000 Abbott Diagnostic or GeneXpert system.

Results

Out of 249 patients with CD who were candidates for biological therapy, 131 (52.6%) received anti-TNFα treatment, including 39 (29.8%) with adalimumab and 92 (70.2%) with infliximab. The median age was 41 years, and the male-to-female ratio was 0.52. The overall HBV screening rate before starting biological therapy was 68.7%. HBV screening was conducted for 90 patients at the CVL, where serological marker analysis categorized five distinct profiles. A majority of patients (65, 72.2%) had negative serological profiles for HBV, while 10 (11.1%) were immunized via vaccination. Profiles at risk of viral reactivation or worsening following immunosuppressive therapy included 12 (13.3%) patients immunized by contact, two (2.3%) with isolated HBcAb, and one (1.1%) with active viral hepatitis (positive HBsAg and HBcAb), who was initiated on tenofovir 300 mg before starting combination therapy. No cases of primary infection or viral B reactivation were observed during the study.

Conclusions

In our study, 15 patients (16.7%) exhibited a potential risk of viral reactivation or worsening of HBV following the initiation of immunosuppressive therapy. The authors recommend precise patient selection, thorough pretreatment evaluation, and regular follow-up during therapy to minimize adverse events associated with anti-TNFα treatment. Additionally, a prophylactic or preemptive strategy should be considered. The risk of late reactivation after discontinuation of biological therapy should also be carefully monitored.

## Introduction

Anti-tumor necrosis factor alpha (TNFα) therapies are monoclonal antibodies that have proven therapeutic efficacy and long-term safety in managing various inflammatory diseases [1]. These therapies have significantly transformed the treatment landscape, enhancing response rates and inducing remission in patients with Crohn’s disease (CD) [2,3]. The antibodies, which are either fully or partially humanized or chimeric IgG1, work by neutralizing TNFα, thereby reducing its serum levels and controlling inflammation and its associated tissue damage [4,5]. Anti-TNFα treatments are administered intravenously or subcutaneously, often in combination with other therapies, following specific regimens for prolonged periods [6].

While these therapies offer substantial benefits, they also pose risks, including immunogenicity and the reactivation of latent infections such as tuberculosis and hepatitis B virus (HBV). The immunosuppressive effects of these treatments can lead to viral reactivation or alter the progression of active HBV infection [7].

Morocco is considered a low-endemicity country for HBV, with an estimated prevalence of 1.8% in the general population [8] and 2.47% among patients at Ibn Sina University Hospital Center (UHC) in Rabat [9]. However, the prevalence of HBV among Moroccan CD patients is not well documented. Furthermore, HBV screening in these patients is not universally practiced, despite existing algorithms [10,11] and recommendations for systematic screening before initiating immunosuppressive therapies [12].

To assess HBV infection, a combination of serological and molecular markers is used. Prior to starting anti-TNFα therapy, three key serological markers should be prioritized: hepatitis B surface antigen (HBsAg), total hepatitis B core antibodies (HBcAb), and hepatitis B surface antibodies (HBsAb). Additional markers, such as HBeAg, HBeAb, IgM HBcAb, and quantitative HBsAg, along with HBV viral load assessment via PCR, may be used for further diagnostic, follow-up, or treatment purposes [10,13,14].

These markers help evaluate a patient’s immune status and identify those at risk for HBV reactivation, as HBV genome persistence (cccDNA) in infected hepatocytes can facilitate reactivation [12]. However, data on the serological status of HBV in Moroccan CD patients receiving anti-TNFα therapy are limited. Therefore, this study aims to evaluate the HBV screening rate before initiating anti-TNFα therapy in CD patients and to assess the prevalence of various serological and molecular profiles, with a particular focus on identifying profiles at risk for HBV reactivation, as well as their follow-up and management.

## Materials and methods

The study was conducted at the Central Virology Laboratory (CVL) in collaboration with the pharmacy department, both affiliated with Ibn Sina UHC in Rabat, Morocco. The patients, primarily from the Rabat-Salé region, were managed by the gastroenterology departments at the same UHC. Data from 249 adult patients with CD, who were candidates for biological therapy, were retrospectively included in the study. Anti-TNFα treatment was initiated at Ibn Sina UHC beginning in January 2015. All patients aged 16 years and older, of both sexes, with CD and for whom anti-TNFα therapy was indicated, were included. Patient records for those considered for anti-TNFα treatment were submitted to the pharmacy department for validation of the therapeutic decision. A multidisciplinary team then developed an optimized management plan for these cases.

For most patients, either as part of the pre-TNFα assessment or during biological therapy, gastroenterologists requested testing for serological and, in some cases, molecular markers of HBV. These tests were performed at the CVL.

The serological profile of each patient included at least HBsAg, HBsAb, and HBcAb, which were assessed using a chemiluminescent microparticle immunoassay on the ARCHITECT i2000sr analyzer or Alinity (Abbott Diagnostics, Chicago, Illinois, United States), in accordance with the manufacturer’s guidelines. Results were expressed in international units per milliliter (IU/ml) for HBsAb and the S/CO ratio for other serological markers. An HBsAb level of 10 IU/L was considered to confer immunity against HBV. Additional tests for HBeAg, HBeAb, and HBc IgM were conducted for HBsAg-positive patients.

For HBsAg-positive patients or those with isolated HBcAb profiles, the viral load was quantified using real-time PCR on the m2000 RealTime System (Abbott Diagnostics) or the Xpert® HBV Viral Load assay on GeneXpert. The detection limit for both systems was 10 IU of DNA/ml of plasma.

The interpretation of different serological profiles in our department (CVL) follows the recommendations of the French National Authority for Health (HAS), as outlined in Table 1 [13].

**Table 1:** Definition of HBV serological profiles based on HBsAg, HBsAb, and HBcAb (according to HAS 2012) ^a^ Occult B infection: A form of chronic HBV infection where HBsAg is undetectable but serum viral DNA levels are typically low. ^b^ Mutations in the S gene, which encodes the envelope viral protein, may lead to the failure of certain diagnostic devices to detect HBsAg. HAS, French National Authority for Health; HBV, hepatitis B virus; HBcAb, hepatitis B core antibodies; HBsAb, hepatitis B surface antibodies; HBsAg, hepatitis B surface antigen

Serological profile			Interpretation
HBsAg-	HBcAb-	HBsAb-	Negative serology HBV
HBsAg-	HBcAb-	HBsAb+	Immunization by vaccination against HBV. Protective level of HBsAb defined by WHO >10 IU/L.
HBsAg-	HBcAb+	HBsAb+	Immunization by contact with the HBV. The patient is currently considered cured, but in some circumstances (severe immunosuppression), reactivation is possible, as in the case of our patients.
HBsAg-	HBcAb+	HBsAb-	Previous contact with HBV with the disappearance of HBsAb. Profile cannot exclude ongoing cure, occult infection^a^, HBsAgb mutant^b^, or a false positive.
HBsAg+	HBcAb-	HBsAb-	Recent acute infection before the appearance of HBcAbs. After confirmation of HBsAg seropositivity by neutralization.
HBsAg+	HBcAb+	HBsAb-	Serological profile suggestive of acute or chronic viral hepatitis B. Is completed with the other markers HBe IgM, HBe IgG, PCR, and quantitative HBsAg. In these isolated cases of HBcAb, HBV PCR should be completed.
HBsAg+	HBcAb+	HBsAb+	HB seroconversion is in progress. Profile cannot exclude chronic infection with the presence of HBsAb (exceptional situation).

Patients’ electronic medical records were reviewed, and data collection was conducted using the “e-LABS” laboratory software and the pharmacy module of the hospital information system. The data gathered included demographic and clinical variables, details of anti-TNFα treatment (type of molecule, dosage, start date, and treatment duration), as well as serological and/or molecular data related to HBV before and during treatment, depending on the patient’s profile. Information on antiviral prophylaxis, if applicable, and any instances of seroconversion were also carefully documented.

Descriptive statistics were presented as the median with IQR for continuous data and as frequency, arithmetic mean, and percentage for categorical variables.

## Results

In our adult cohort of CD patients, biological therapy was considered the appropriate treatment for 249 individuals. Among these, 131 patients received anti-TNFα therapy, with 92 (70.2%) treated with infliximab (IFX) and 39 (29.8%) with adalimumab (ADA).

**Figure 1:**
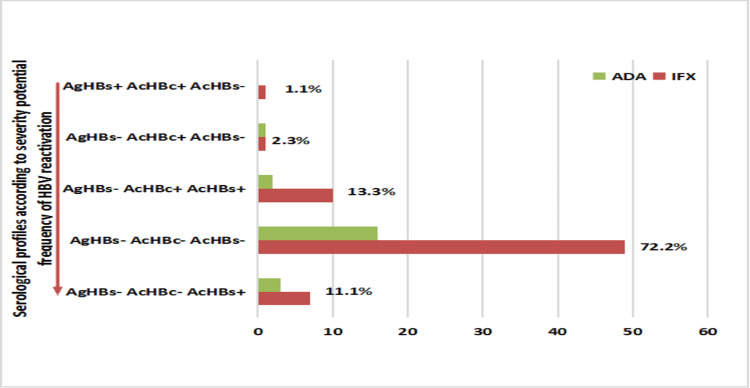
Number and percentage of different hepatitis B profiles in CD patients on anti-TNFα at Ibn Sina UHC in Rabat CD, Crohn’s disease; TNFα, tumor necrosis factor alpha; UHC, University Hospital Center

Baseline characteristics and HBV status of the CD patients prior to initiating anti-TNFα therapy are summarized in Table 2 and Figure 1. The median age of the patients was 41 years (range: 27-50), and the male-to-female ratio (M/F) was 0.52. The HBV screening rate before starting biological therapy was 68.7% (90/131 patients), with a progressive increase from 38.1% in 2016 to 92.5% in 2022. The median duration of anti-TNFα therapy was 825 days (range: 300-1095 days). Of the patients, 49 (55%) received anti-TNFα monotherapy, while 41 (45%) underwent combination therapy.

**Table 2:** Characteristics of patients with CD on anti-TNFα ADA, adalimumab; CD, Crohn’s disease; HBV, hepatitis B virus; IFX, infliximab; TNFα, tumor necrosis factor alpha

Population characteristics	Treatment	Value
Recruited patients (n)		131
Male sex		45 (34.3%)
Age (median, IQR)		41 (27.50)
Anti-TNFα	ADA 40 mg inj	39 (29.8%)
	IFX 100 mg inj	92 (70.2%)
Duration of treatment anti-TNFα (median, IQR)		825 j (300-1,095)
Prophylaxis of HBV infection	Tenofovir 300 mg cp	1 (0.88%)

The combination agents used alongside anti-TNFα therapy included azathioprine (AZA), methotrexate, and 6-mercaptopurine. Analysis of the serological markers in the 90 CD patients receiving anti-TNFα therapy revealed five distinct HBV serological profiles (Table 3).

**Table 3:** Serological profiles of HBV infection in our series (90 patients) HBV, hepatitis B virus; HBcAb, hepatitis B core antibodies; HBsAb, hepatitis B surface antibodies; HBsAg, hepatitis B surface antigen

HBV infection	Serological profiles	Value
Chronic hepatitis B	HBsAg+ HBcAb+ HBsAb-	1 (1.1%)
Isolated HBcAb	HBsAg- HBcAb+ HBsAb-	2 (2.3%)
Contact by immunization	HBsAg- HBcAb+ HBsAb+	12 (13.3%)
Immunization by vaccination	HBsAg- HBcAb- HBsAg+	10 (11.1%)
Negative serology of HBV	HBsAg- HBcAb- HBsAb-	65 (72.2%)

In decreasing order of potential risk for HBV reactivation, the following profiles were identified (Figure 1): One patient (1.1%) exhibited a chronic hepatitis B profile (HBsAg+, HBcAb+, HBsAb-) with positive HBeAb and a viral load of 2.9 log IU/ml and received anti-HBV therapy (tenofovir 300 mg) before starting IFX in combination with AZA. Two patients (2.3%) presented with isolated HBcAb status (HBsAg-, HBcAb+, HBsAb-), while 12 patients (13.3%) were immunized through past HBV exposure (HBsAg-, HBcAb+, HBsAb+). A predominance of negative serological status (HBsAg-, HBcAb-, HBsAb-) was observed in 65 patients (72.2%). Among 10 patients (11.1%) vaccinated against HBV, a protective immune response (HBsAg-, HBcAb-, HBsAb+) was noted, with no risk of HBV infection; however, two patients lost their initial HBsAb titers, which ranged from 10 IU/L to 100 IU/L, during biological therapy.

In 9.16% of cases, a therapeutic switch from IFX to ADA was required due to adverse effects, including allergic reactions, anaphylactic shock, psoriasis, or the development of anti-TNFα antibodies. Treatment optimization through dose escalation was applied in 5.9% of cases, while anti-TNFα therapy was discontinued in 7.6% of patients due to complications such as tuberculosis reactivation, thymoma, or death.

## Discussion

The natural history of HBV infection, the progressive nature of CD, and the use of novel therapies, such as anti-TNFα, carry risks, including HBV reactivation, which can affect both hepatic and intestinal outcomes [6,13,15]. In our study, the overall HBV screening rate before starting anti-TNFα therapy was 68.7%, showing an upward trend over the years, but this rate is lower than the 82.3% reported by Fidan et al. [7]. International guidelines recommend systematic serological screening prior to initiating anti-TNFα treatment [16-18]. The risk of reactivation is stratified based on serovirological status and the type of immunosuppressant used.

Regarding serovirological status, the reactivation risks in decreasing order are detectable HBV DNA, HBsAg+ status, HBeAg+ status, and HBcAb+ status [15]. In our study, 15 patients (16.7%) were identified as being at potential risk for viral reactivation or worsening HBV infection. One patient (1.1%) with an HBsAg+/HBcAb+ profile, indicative of chronic HBV infection, tested negative for HBeAg. The HBsAg level was 4800.77 S/CO, and HBeAb was positive. This patient, diagnosed with ileocolic Crohn’s stenosing phenotype, had a viral load of 811 IU/ml (2.9 log IU/ml). Liver function tests were normal, and the patient, a candidate for combination therapy (IFX + AZA), received preemptive HBV treatment with tenofovir (300 mg/day). After one month, HBV DNA levels became undetectable.

For patients with HBsAg (either active chronic hepatitis or inactive carriers), HBV DNA quantification is essential. Preemptive therapy with nucleos(t)ide analogs is recommended, followed by regular monitoring (transaminases and viral load every one to three months). If anti-TNFα therapy is discontinued, preemptive therapy should continue for six to 12 months [19,20].

An Italian multicenter study [21] reported a higher prevalence of HBcAb in CD patients compared to controls, whereas a similar Spanish study [22] did not. In our study, two patients had isolated HBcAb status, one on IFX and the other on ADA, both with undetectable viral loads before anti-TNFα treatment and no declared viral reactivation. The prevalence of isolated HBcAb in CD patients was 2.3%, comparable to 1.81% in the general Moroccan population [8], but lower than the 4.60% observed in the general hospital population at Ibn Sina UHC [9].

The rate of HBV reactivation during anti-TNFα therapy for immune-mediated inflammatory diseases is generally low [6]. However, the American Gastroenterological Association (AGA) recommends antiviral prophylaxis for all HBcAb+ patients, regardless of HBsAg status [23]. In contrast, the European Crohn’s and Colitis Organization suggests viral load monitoring every two to three months for HBcAb+/HBsAg- patients, with antiviral prophylaxis only if HBV DNA is detected [24].

In HBcAb+/HBsAg- patients, viral DNA detection requires highly sensitive assays due to the low concentration of HBV DNA in plasma (<200 IU/ml) [25,26]. Occult HBV infection should be considered in all HBcAb+/HBsAg- patients [25]. The first case of occult hepatitis B reactivation in a CD patient treated with IFX was reported by reference [27].

No specific guidelines exist for initiating antiviral therapy in CD patients with occult HBV who are candidates for biologic therapy [20]. In our study, 13.3% of patients had an HBsAg-/HBcAb+/HBsAb+ profile, indicating resolved HBV infection. One study by Lee et al. [28] reported hepatic dysfunction linked to viral B reactivation in an inflammatory bowel disease patient with this “cured” status. In contrast, a meta-analysis of rheumatology cases reported a low (1.7%) risk of HBV reactivation in patients with resolved infection, provided HBsAb titers are maintained at protective levels [29]. Monitoring transaminases, HBsAg, and viral load is recommended, without the need for systematic preemptive treatment [17].

For the majority of patients with an HBsAg-/HBcAb-/HBsAb- profile (72.2%), HBV vaccination is strongly recommended by the European Association for the Study of the Liver and other international bodies [13,30,31]. However, the response rate to vaccination in CD patients, particularly those treated with IFX, is often low [32-35]. In our series, two IFX-treated patients lost their HBsAb titers during treatment. Studies by Jian et al. and Gisbert et al. [32,33] suggest that response rates can be improved with an accelerated, double-dose vaccine regimen. Seroprotection against HBV infection was redefined in these patients at a concentration ≥100 IU/l [33]. Consequently, the HBV vaccine should be administered prior to biological therapy initiation and during the stabilization phase of the disease [30].

For patients with high HBsAb levels before treatment, the risk of HBV-related complications is low, and antiviral prophylaxis may not be necessary. No cases of primary infection or HBV reactivation were observed in this study, consistent with findings by William et al., who reported a low reactivation rate in HBsAg-/HBcAb+ patients treated with anti-TNFα [6].

Anti-TNFα therapy is the standard treatment for CD refractory to corticosteroids and immunosuppressants. The AGA considers the risk of HBV reactivation with TNFα inhibitors to be intermediate (1-10%) [36,37]. Studies have shown that TNFα plays a key role in regulating HBV replication, and blocking this pathway can lead to HBV reactivation [28,38].

HBV reactivation is defined as a rise of at least 1 log10 IU/ml in serum HBV DNA or the sudden appearance of HBV replication in patients with inactive or resolved hepatitis B, along with an increase in serum ALAT [39].

Regarding the nature of the immunosuppressant, IFX is more frequently associated with HBV reactivation than ADA [40,41], with cases of fulminant hepatitis reported in CD patients treated with IFX [42,43]. One case illustrates fulminant acute hepatitis B in a patient with CD treated with combination therapy (IFX/AZA), which was fatal despite the initiation of lamivudine [44]. Perrillo [17] has proposed an algorithm for managing patients planned for anti-TNFα therapy. Entecavir and tenofovir are recommended for prophylaxis due to their high resistance barrier [45,46].

A limitation of this study is that patients who had serological tests performed outside the CVL may not have been included in the data.

## Conclusions

This study highlights HBV screening rates and serological profiles in CD patients treated with anti-TNFα. Screening rates have increased over time, and no HBV reactivation with HBsAg seroconversion was observed. However, a notable percentage of patients presented with potential risk factors for viral reactivation, emphasizing the importance of thorough evaluation before therapy initiation and regular monitoring during treatment. Systematic screening for HBV markers and vaccination for HBV-negative patients are crucial. Moreover, patients discontinuing treatment should undergo periodic serological monitoring to mitigate the risk of late reactivation.
